# Diversity in Emotional Support, and Its Social, Historical, and Age-Related Trends

**DOI:** 10.1093/geroni/igaf122.1606

**Published:** 2025-12-31

**Authors:** Erin Clancy, Rachel Koffer

**Affiliations:** Arizona State University, Phoenix, Arizona, United States; Arizona State University, Phoenix, Arizona, United States

## Abstract

In this study, we introduce the concept of emotional support diversity: the breadth and balance of emotional support given and received from different individuals in one’s social network. Further, we test how developmental (i.e., age), historical (i.e., birth cohorts), and social (i.e., sexual minority status) contexts relate to differences in emotional support diversity. We utilized data from two independent cohorts of the Midlife in the United States (MIDUS) study (1995/1996 cohort: n = 5,492 adults aged 24-75 years; 2013/2014 cohort: n = 2,495 adults aged 23-76, stratified by age and gender to match the 1995-96 cohort). An overall emotional support diversity score was calculated based on 6 social roles (e.g., spouse or partner, in-laws) across 2 pathways (i.e., giving and receiving emotional support) using the Gini index. General linear models controlling for gender, race, education, self-rated health, and network size (i.e., partnership and parental status), revealed that older adults reported less emotional support diversity (b = -0.005, p < .001). The later-born cohort experienced less overall emotional support diversity (b = -0.022, p = 0.035). An interaction between age and cohort effects revealed that those in early midlife experienced less overall emotional support diversity in the later-born cohort than in the earlier-born cohort (b = 0.002, p = 0.025). There were no differences in emotional support diversity by sexuality (ps >.05). Implications of this new construct and differences in the hours of support received and given by specific social roles will be discussed in the context of age, cohort, and sexual minority status.

